# Supplementary material for: Bidirectional crosstalk between Hypoxia-Inducible Factor and glucocorticoid signalling in zebrafish larvae
Source: PLoS Genet. 2020 May 7;16(5):e1008757. doi: 10.1371/journal.pgen.1008757 (PMC7237044; doi:10.1371/journal.pgen.1008757)
Supplement: Attachment — Submitted filename: review.docx [file pgen.1008757.s007.docx]

In this manuscript, the authors have studied the relationship between the HIF and glucocorticoid signaling pathways, using the zebrafish as an in vivo model system. This study is a follow-up of a previous paper (Vettori et al., PNAS 2017), in which they showed that glucocorticoids enhance HIF signaling through stabilization of pVHL. The authors make use of a *vhl* mutant line and have generated an *arnt1* and a *gr* mutant line, and generated G0 CRISPants to knock out *mr* and *arnt2*. Gr was activated by betamethasone treatment. As readouts for Gr activity, the expression of several target genes was measured by qPCR or WISH. To determine HIF activity, the phd3:GFP reporter line was used, as well as qPCR and WISH of target genes. To study effects on cortisol biosynthesis, whole body cortisol levels were measured.

The results show that the responsiveness to glucocorticoids is decreased when HIF signaling is increased and vice versa. Interestingly, in line with a previously published study (Tan et al., 2017), the authors show decreased cyp17a2 mRNA levels upon increased HIF signaling, and cortisol levels are similarly regulated. A surprising result is the decreased pomca expression in both the vhl and the arnt1 mutants (with opposite effects on HIF signaling). Furthermore, the authors show that decreased Gr signaling enhances activated HIF signaling (gr mutation partially rescues vhl mutant phenotype, and even further rescues the vhl;arnt1 mutant phenotype, whereas BME increases target gene expression in vhl mutant (not in vhl;arnt1 mutants)). Interestingly, vhl mutation could not override the strong upregulation of pomca in the gr mutant. Finally, the authors demonstrate that Mr activity has a similar (additive) effect on HIF signaling as GR activity.

This manuscript reveals an intricate network of interactions between glucocorticoid and HIF signaling, two major signaling pathways. These interactions have large consequences for the response to (hypoxic) stress and possibly for the efficacy of glucocorticoid treatment of immune-related diseases. Technically, the study looks well performed using CRISPR/Cas9 gene editing as one of the main research tools. The available data therefore look sound. However, I have three major concerns:

1) While studying the interaction between glucocorticoid and HIF signaling, the authors are dealing with three types of interactions: i) The HIF effect on glucocorticoid responsiveness (measured by BME effect on target gene expression); ii) The HIF effect on steroid biosynthesis (measured by expression of pomca, cyp17a and cortisol level in vhal and arnt1 mutant larvae); iii) The glucocorticoid effect on HIF signaling (measured by phd:GFP reporter line and expression of target genes in gr mutant larvae and after BME treatment). As a result, the manuscript lacks a clear focus, and is difficult to read, especially the second half of the Results section (where text and figures are not following the same order) and in the Discussion. I would suggest the authors to reconsider the structure of the paper to disentangle these three questions. For example, one could first introduce the novel mutant lines (arnt-/ and gr -/-) and then present the data by separating the readouts (and thus the 3 questions).
2) In both the Results section and the Discussion, I think the authors confuse HIF effects on glucocorticoid responsiveness and on cortisol biosynthesis. Sometimes it seems like they are considered as one phenomenon and sometimes as being in the same linear pathway (Figs.3B’and C’, and Discussion (p.15)). However, I think they should be considered as separate (though interfering) effects. It is highly exceptional and counterintuitive that a glucocorticoid-resistant condition coincides with low cortisol levels (as observed in the vhl mutant). Generally, high cortisol levels are seen as a hallmark of glucocorticoid resistance (as e.g. in the gr mutant fish). This can in my opinion only be explained by HIF signaling enhancing Gr responsiveness and additionally having direct repressive effects on genes involved in cortisol biosynthesis (which should not be confused with negative feedback as in Fig.3C’).

Interestingly, these two effects interfere at the level of pomca expression, which is subject to suppression by HIF and glucocorticoid signaling (the latter being negative feedback). The WISH results show a decrease in expression levels in both the the vhl (increased HIF signaling) and the arnt1 (decreased HIF signaling) mutants. It may be suggested that HIF directly suppresses the pomca expression (just like other genes involved in cortisol production) in the vhl mutant. The decrease in the arnt-/- mutant could be explained by a combination of the higher cortisol levels and the increased Gr responsiveness (as determined by BME treatment). The authors are encouraged to rewrite the interpretation of their results along these lines.

3) The authors have chosen to use zebrafish larvae as the model system in this study. This provides the opportunity to study the role of the two signaling pathways in an intact organism, but it is questionable if this opportunity is fully exploited. For example, in the Discussion the authors speculate about the physiological relevance of their findings in the (hypoxia) stress response, and I would suggest that the authors test their hypothesis by exposing larvae to hypoxia stress. In addition, one could argue that most results could have been obtained in cell cultures as well, which would have given the opportunity to study certain molecular mechanism in more detail. For example, I would be interested to see how HIF enhances the Gr responsiveness of the studied target genes (through tethering/acting as a transcriptional coactivator?) , how HIF suppresses the pomca transcription (direct binding to the promoter?), and how Gr enhances HIF signaling in the absence of Vhl.

Minor concerns:

- The MR and GR as two receptors for cortisol with different affinities should be mentioned in the first paragraph of the Introduction.
- HIF is not a member of the nuclear receptor family, as suggested in the Introduction.
- In the Introduction, the nomenclature of zebrafish and mammalian HIF subunits should be explained. HIF, HIF-1, HIF-alpha (also used in Discussion) and HIF-1alpha are used without making clear what the difference is, and it becomes even more complicated when zebrafish nomenclature is introduced. How do the zebrafish Hif proteins relate to their mammalian equivalents?
- I think that the term ‘attenuated’ is misleading when used for the HIF signaling in the double vhl;arnt mutant. It only shows attenuated HIF signaling in comparison to the vhl mutant, but compared to wild type the HIF signaling is actually increased.
- In Figs. 2A, 3C’, and S2, the names of the mutants should be vhl-/- and arnt1-/-, just like in the other figures.
- In Fig.2A and S2, large differences are observed in the responsiveness to BME in the siblings. Shouldn’t they show the same response? If not, wild type controls should be added.
- It is not clear why the expression of vegfab and egln3 are measured (Fig.3C’). Please mention that they are HIF target genes.
- It is not well defined what a ‘proper’ HIF response is. I think the authors should refrain from using this term, since its meaning is unclear.
- Figures 6Cand 6D could be supplemental figures.
- Minor textual issues:
  - At many points in the manuscript, the authors leave out the articles (‘the’ and ‘a/an’). For example, it should be ‘the response’, ‘the phenotype’, ‘the pathway’.
  - P.6, line 6: ‘arnt’ instead of ‘artn’
  - P.7: ‘fold change=0.1’ instead of ‘0,1’. This should be corrected throughout this paragraph.
  - P.12: Please correct the sentence: ‘To confirm the specificity of Wu et al., 2018 method,…’
  - P.14: The abbreviation ‘WISH’ has already been introduced.
  - P.14, line 4: ‘suggest’ instead of ‘suggests’
  - P.15: the abbreviation ‘GC’ should be consistently used throughout the manuscript and introduced in the Introduction (not just in the Abstract). It should be ‘GC-induced’, and not ‘GCs-induced’ (and likewise ‘GC target genes’ and ‘GC response’ elsewhere in the manuscript).
