# Supplementary material for: Bidirectional crosstalk between Hypoxia-Inducible Factor and glucocorticoid signalling in zebrafish larvae
Source: PLoS Genet. 2020 May 7;16(5):e1008757. doi: 10.1371/journal.pgen.1008757 (PMC7237044; doi:10.1371/journal.pgen.1008757)
Supplement: Attachment — Submitted filename: Response to reviwers_DMFvE_07-02-2020.docx [file pgen.1008757.s008.docx]

Please see below the itemized response to reviewers (highlighted in red):

**Reviewer #1:**

In this manuscript, the authors have studied the relationship between the HIF and glucocorticoid signaling pathways, using the zebrafish as an in vivo model system. This study is a follow-up of a previous paper (Vettori et al., PNAS 2017), in which they showed that glucocorticoids enhance HIF signaling through stabilization of pVHL. The authors make use of a *vhl* mutant line and have generated an *arnt1* and a *gr* mutant line, and generated G0 CRISPants to knock out *mr* and *arnt2*. Gr was activated by betamethasone treatment. As readouts for Gr activity, the expression of several target genes was measured by qPCR or WISH. To determine HIF activity, the phd3:GFP reporter line was used, as well as qPCR and WISH of target genes. To study effects on cortisol biosynthesis, whole body cortisol levels were measured.

The results show that the responsiveness to glucocorticoids is decreased when HIF signaling is increased and vice versa. Interestingly, in line with a previously published study (Tan et al., 2017), the authors show decreased cyp17a2 mRNA levels upon increased HIF signaling, and cortisol levels are similarly regulated. A surprising result is the decreased pomca expression in both the vhl and the arnt1 mutants (with opposite effects on HIF signaling). Furthermore, the authors show that decreased Gr signaling enhances activated HIF signaling (gr mutation partially rescues vhl mutant phenotype, and even further rescues the vhl;arnt1 mutant phenotype, whereas BME increases target gene expression in vhl mutant (not in vhl;arnt1 mutants)). Interestingly, vhl mutation could not override the strong upregulation of pomca in the gr mutant. Finally, the authors demonstrate that Mr activity has a similar (additive) effect on HIF signaling as GR activity.

This manuscript reveals an intricate network of interactions between glucocorticoid and HIF signaling, two major signaling pathways. These interactions have large consequences for the response to (hypoxic) stress and possibly for the efficacy of glucocorticoid treatment of immune-related diseases. Technically, the study looks well performed using CRISPR/Cas9 gene editing as one of the main research tools. The available data therefore look sound.

However, I have three major concerns:

1) While studying the interaction between glucocorticoid and HIF signaling, the authors are dealing with three types of interactions: i) The HIF effect on glucocorticoid responsiveness (measured by BME effect on target gene expression); ii) The HIF effect on steroid biosynthesis (measured by expression of pomca, cyp17a and cortisol level in vhal and arnt1 mutant larvae); iii) The glucocorticoid effect on HIF signaling (measured by phd:GFP reporter line and expression of target genes in gr mutant larvae and after BME treatment). As a result, the manuscript lacks a clear focus, and is difficult to read, especially the second half of the Results section (where text and figures are not following the same order) and in the Discussion. I would suggest the authors to reconsider the structure of the paper to disentangle these three questions. For example, one could first introduce the novel mutant lines (arnt-/ and gr -/-) and then present the data by separating the readouts (and thus the 3 questions).

1) **Our response:** We acknowledge that the second half of the results and of the discussion section could have been difficult to read. For this reason, we have improved them by rephrasing unclear concepts and increasing the amount of subpanels designators. Moreover, the complexity both of data and the text have been improved by removing the moderate upregulated HIF levels’ RTqPCR data on fkbp5 expression (central graph in figure 2A). As a consequence of the above modifications, we consider the current version of the paper easier to read and to understand.

2) In both the Results section and the Discussion, I think the authors confuse HIF effects on glucocorticoid responsiveness and on cortisol biosynthesis. Sometimes it seems like they are considered as one phenomenon and sometimes as being in the same linear pathway (Figs.3B’and C’, and Discussion (p.15)). However, I think they should be considered as separate (though interfering) effects. It is highly exceptional and counterintuitive that a glucocorticoid-resistant condition coincides with low cortisol levels (as observed in the vhl mutant). Generally, high cortisol levels are seen as a hallmark of glucocorticoid resistance (as e.g. in the gr mutant fish). This can in my opinion only be explained by HIF signaling enhancing Gr responsiveness and additionally having direct repressive effects on genes involved in cortisol biosynthesis (which should not be confused with negative feedback as in Fig.3C’). Interestingly, these two effects interfere at the level of pomca expression, which is subject to suppression by HIF and glucocorticoid signaling (the latter being negative feedback). The WISH results show a decrease in expression levels in both the vhl (increased HIF signaling) and the arnt1 (decreased HIF signaling) mutants. It may be suggested that HIF directly suppresses the pomca expression (just like other genes involved in cortisol production) in the vhl mutant. The decrease in the arnt-/- mutant could be explained by a combination of the higher cortisol levels and the increased Gr responsiveness (as determined by BME treatment). The authors are encouraged to rewrite the interpretation of their results along these lines.

2) **Our response:** We entirely agree that GC responsiveness and cortisol biosynthesis should be considered as separate (though interfering) effects. To this end, we modified it accordingly both in the Results and the Discussion sections. However, even if it is rare and counterintuitive that a glucocorticoid-resistant condition coincides with low cortisol levels, we speculate that HIF signalling can act as a second controller of the GC-mediated stress response (in addition to cortisol levels themselves). We have observed a strong downregulation of *fkbp5* in *vhl* mutants compared to *vhl* siblings (Fig. 2A). Vice versa, when *arnt1* is mutated in a wildtype background a significant upregulation of *fkbp5* has been observed in *arnt1* mutants compared to *arnt1* siblings (Fig. 2A’). This allowed us to assume that if GC can enhance HIF signalling (Vettori et al 2017), in turn, HIF is able to indirectly control both HIF levels themselves and the GC ones, by acting on *pomca*. This would enable HIF signalling to assure both stress resolution and homeostasis. Moreover, since GC have an anti-inflammatory roles and HIF act as a pro-inflammatory factor, the simultaneous upregulated expression of both pathways would be detrimental to homeostasis itself.

Finally, we entirely agree with your stamentent that “HIF directly suppresses the pomca expression (just like other genes involved in cortisol production) in the *vhl* mutant. The decrease in the *arnt1-/-* mutant could be explained by a combination of the higher cortisol levels and the increased Gr responsiveness (as determined by BME treatment)” (page 16 line 10-32).

3) The authors have chosen to use zebrafish larvae as the model system in this study. This provides the opportunity to study the role of the two signaling pathways in an intact organism, but it is questionable if this opportunity is fully exploited. For example, in the Discussion the authors speculate about the physiological relevance of their findings in the (hypoxia) stress response, and I would suggest that the authors test their hypothesis by exposing larvae to hypoxia stress. In addition, one could argue that most results could have been obtained in cell cultures as well, which would have given the opportunity to study certain molecular mechanism in more detail. For example, I would be interested to see how HIF enhances the Gr responsiveness of the studied target genes (through tethering/acting as a transcriptional coactivator?) , how HIF suppresses the pomca transcription (direct binding to the promoter?), and how Gr enhances HIF signaling in the absence of Vhl.

3) **Our response:** We acknowledge your point, but unfortunately it is not possible to study the intricate HIF-GC crosstalk *in vitro* as efficiently as *in vivo.* Indeed, the Hypothalamus-pituitary-interrenal axis, with its GC-GR mediated negative feedback loop are not present in single cells. Moreover, it is only thanks to the use of the animal model used in this study that has been possible to highlight the ability of GC to stimulate HIF pathway and consequently the HIF signalling capacity to control *pomca* expression to assure homeostasis. Finally, we have not attempted to expose larvae to real hypoxia stress. Unfortunately, in contrast to young embryos older larvae (which we require for our analysis) are quite sensitive to prolonged hypoxia and quickly become sick and die. For this reason, only by mutating *vhl* or *arnt1* gene it is possible to effectively either upregulate or downregulate HIF pathway respectively.

4) Minor concerns:

- The MR and GR as two receptors for cortisol with different affinities should be mentioned in the first paragraph of the Introduction.

**Our response:** We have amended the manuscript as suggested (page 2, line 29-31)

- HIF is not a member of the nuclear receptor family, as suggested in the Introduction.

**Our response:** We apologise, this has been corrected.

- In the Introduction, the nomenclature of zebrafish and mammalian HIF subunits should be explained. HIF, HIF-1, HIF-alpha (also used in Discussion) and HIF-1alpha are used without making clear what the difference is, and it becomes even more complicated when zebrafish nomenclature is introduced. How do the zebrafish Hif proteins relate to their mammalian equivalents?

**Our response:** We apologise for not making this clear, this has been corrected. (page 3, line 7-15)

- I think that the term ‘attenuated’ is misleading when used for the HIF signaling in the double vhl;arnt mutant. It only shows attenuated HIF signaling in comparison to the vhl mutant but compared to wild type the HIF signaling is actually increased.
- **Our response:** We have amended the manuscript as suggested. Now, the term attenuated has been changed into moderately upregulated throughout the text.
- In Figs. 2A, 3C’, and S2, the names of the mutants should be vhl-/- and arnt1-/-, just like in the other figures.

**Our response:** We apologise for this oversight. This has been corrected.

- In Fig.2A and S2, large differences are observed in the responsiveness to BME in the siblings. Shouldn’t they show the same response? If not, wild type controls should be added.

**Our response:** No, we think this is not that surprising because of variability between clutches of embryos. Moreover, other factors such as the larval developmental stage and the fixation time of the embryos may slightly differ/not always perfectly match. This could result in a slightly different GC responsiveness observed in the different group of siblings. For this reason, we speculate that the use of wt controls will face the same aforementioned issues.

- It is not clear why the expression of vegfab and egln3 are measured (Fig.3C’). Please mention that they are HIF target genes.

**Our response:** We apologise for not making this clear. This has been corrected (page 11, line 29).

- It is not well defined what a ‘proper’ HIF response is. I think the authors should refrain from using this term, since its meaning is unclear.

**Our response:** We acknowledge that proper could an ambiguous term. For this reason, we have removed the term ‘proper’ from the text as suggested.

- Figures 6Cand 6D could be supplemental figures.

**Our response:** We have moved the two figures in the supplementary data, as suggested. Now they can be found as supplementary Fig S6.

- Minor textual issues:
  - At many points in the manuscript, the authors leave out the articles (‘the’ and ‘a/an’). For example, it should be ‘the response’, ‘the phenotype’, ‘the pathway’.
  - P.6, line 6: ‘arnt’ instead of ‘artn’
  - P.7: ‘fold change=0.1’ instead of ‘0,1’. This should be corrected throughout this paragraph.
  - P.12: Please correct the sentence: ‘To confirm the specificity of Wu et al., 2018 method…’
  - P.14: The abbreviation ‘WISH’ has already been introduced.
  - P.14, line 4: ‘suggest’ instead of ‘suggests’
  - P.15: the abbreviation ‘GC’ should be consistently used throughout the manuscript and introduced in the Introduction (not just in the Abstract). It should be ‘GC-induced’, and not ‘GCs-induced’ (and likewise ‘GC target genes’ and ‘GC response’ elsewhere in the manuscript).

**Our response:** We accept all these points and have addressed them accordingly.

**Reviewer #2**

The manuscript by Marchi et al, “Bidirectional crosstalk between Hypoxia-Inducible Factor and glucocorticoid signaling in zebrafish larvae” provides new information about the crosstalk of hypoxia and glucocorticoid signaling in vivo.

In addition to the scientific conclusions presented, the authors have produced another GR (nr3c1) mutant allele (currently 9 listed on [zfin.org](http://zfin.org)) and a new HIF1B (arnt) mutant (5 listed on ZFIN). Many of the nr3c1 alleles are similar to the new allele, but potentially published after initiation of this work. The arnt alleles represent mass deposited alleles from Sanger or the NIH, which likely were not studied, and are therefore not certain loss of function or nulls. My primary concern is that the organization and presentation of the data in the current version of this manuscript is cumbersome. The figures have multiple panels that when printed are not legible as the fonts are far too small. In some cases they are even hard to see on my monitor zoomed to greater than 200%. This made the key elements of this paper difficult to readily grasp. However, the key findings are important. With a significant overhaul of presentation, including perhaps rethinking figure organization would greatly improve both the impact and reach of the science presented by the authors. Below I have listed some additional major/minor concerns.

**Our response:** We apologise and we have rethought figure organization. Moreover, we have increased both the number of subpanels for each figure and the size of the fonts too. Figures are now bigger than before and easier to read. We have also rephrased complex concepts in a simpler way and have added more links between the text and the figures. Finally, we confirmed that our *arnt1* mutant line is a true null as it was able to significantly downregulate both the expression of our hypoxia reporter (*phd3:eGFP*) and of the endogenous *egnl3* expression as observed via phenotypic analysis and/or RTqPCR (Figure 1E-F,K and S1C).

Other Major concerns:

QRT data shows mixed results (by gene target) which are not brought into the model or discussion. Why is BME, a GR agonist not able to increase expression of GR target genes? For some of these targets the mutant background appears to change basal expression levels and the BME still increases expression but not to the same degree as in wild type. Is the ratio of the increase from baseline to BME rates similar? This is difficult to see in figures.

**Our response:** We initially used a set of genes that have been published in other organisms as targets of the GR pathway, however whether this is true in whole zebrafish larvae at the stage where we are analysing our results was not clear, also the extent of the response may be low. For this reason, we have now focused our result on just two genes (*fkbp5* and *pomca*) that are well-established as GR readout at this stage, and have been accepted as readouts in other papers (Binder, 2009; Griffiths *et al.*, 2012; Ziv *et al.*, 2014; Lin *et al.*, 2015; Facchinello *et al.*, 2017) and also have a high responsiveness in terms of FC or ISH changes.

Figure 2F and genotyping of mutants. The methods do not detail how mutants were genotyped. To do the cortisol assays in 2F the genotype of the larvae would have to be known prior to collection as a group for the ELISA. How was this done, is this indeed basal cortisol or acute stress, etc. (Minor) why is this expressed per 150 embryos on the y-axis?

**Our response:** Steroids have been extracted and basal cortisol levels has been quantified as described in the material and method section. In particular, cortisol quantification was carried out according to the protocol published by Eachus et al., 2017 (Eachus et al., 2017), based on the use of an Acquity UPLC System (Waters, Milford, CT) coupled to a Xevo TQ-S tandem mass spectrometer (Waters), and not on the ELISA method. Moreover, *vhl* -/- larvae were sorted from *vhl* siblings (prior to collection at 4 dpf) according to their easily recognizable *phd3:eGFP* expression. On the other hand, because of the lack of visible phenotype, *arnt1-/-* larvae derived from *arnt1-/-* fish incrossed, whereas siblings from *arnt1+/-* fish crossed with *arnt1+/+* ones. Finally, we modified the graph by using fg/larva on the y axis instead of pg/150 larvae as suggested. We previously used pg/150 larvae because it was the amount of larvae present in each of the three biological replicates used for cortisol extraction and quantification. (page 21, line 24-32).

Figure 3B image is poor, data is not new per se so could be mentioned (not shown) or moved to supplemental to make room for key novel data. Fig 3B’ these images do not readily demonstrate changes in expression.

**Our response**: We have removed figure 3B as suggested. We have also modified the figure 3B’ (now referred as figure 3B-C’) by adding a magnified picture of the area of interest to show the upregulation of pomca at the level of the anterior part of the pituitary gland. This occur when there is no functional Gr to assure a proper GC-Gr mediated negative feedback loop. Finally, we confirmed that by quantifying *fkbp5* expression (GR activity) via RTqPCR analysis.

Loss of MR in crispant appears to more severely impact HIF-reporter line than GR. How well do the guides target MR in Crispants (no molecular data is shown for Crispant efficiency)? How does the model reflect or change with the possibility that MR is potentially a major mediator of HIF:GC interactions, rather than GR? This is a really interesting observation that would be better studied in a MR mutant background however, I do not think that is a reasonable ask your manuscript has provided a lot of great data. However, discussing this outcome and possible meaning for future research is necessary.

**Our response**: CRISPR gRNAs’ sequences are now listed at page 20, line 14-17. We observed a strong downregulation of the hypoxia reporter especially mainly at the level of the head of *mr* injected *vhl-/-* compared to *gr-/-;vhl-/-* (page 17 line 33, and Fig 6D-B and 6G)*.* For this reason, we believe that Mr plays a pivotal role in the head, whereas Gr mainly acts in the rest of the body. Nevertheless, we completely agree with you that this is still a really interesting observation that would be better studied in a Mr mutant background. We also believe that Mr activity perfectly fits with our model. Indeed, Mr acts at the same level of Gr (GC-response in Fig 2G) and it can bind to cortisol, even if with different affinity. Moreover, when both Gr and Mr were knocked-out HIF signalling was further suppressed compared to *gr;vhl* double mutants.

Minor comments:

The authors use a HIF-reporter line, eGFP driven by the phd3 promoter, and a previously published Von Hippel Lindau (VHL) mutant that increases HIF function and activates EGFP in the HIF-reporter line. When scoring GFP changes they quantify within the head, liver, and tail. A single image showing how these regions were scored would be helpful.

**Our response**: We really appreciate this advice. We have added it as the supplementary figure S1D (page 43).

In both Figure 1 and Figure 2 the gene map is presented in reverse (as if looking at a whole genome map). However, there is no reason not to orient the single genes from 5’ to 3’ in these figures. For Figure 2 the arrow that indicates the mutation site is pointing at exon 4 and not exon 3.

**Our response**: We apologise, these have been corrected.

There could be more subpanel designators in figures. For example, Figure 1 has insets without designators, and this makes it more difficult to find what the author is referencing in the text.

**Our response**: We apologise for that. We have amended the manuscript as suggested, by adding more insets and designators to improve the readability.

There are some locations in the text and figures (including supplemental) that use hif1ßsh544, arnt1sh544 and at least one artn1sh544.

**Our response**: We apologise for this oversight, this has been corrected.

In Figure 2A and FigS2 the Y-axes range is large due to BME treatment and thus it becomes difficult to see physiological response in the absence of GR agonist.

**Our response**: We apologise, this has been corrected to improve the readability.

In situ hybridization is difficult to get quantitative data from, though qualitatively as shown it is observed to be higher or lower expression. Could QRT of the same target genes (pomc and cyp17a2) provide better quantification?

**Our response**: Unfortunately, we do not think it could. This is due to the minuscule size of both the pituitary and the interrenal gland which makes really difficult to reliably quantify both *pomca* and *cyp17a2* expression via RTqPCR.

Figure 3C. No direct comparison to vhl-/-, which is in other locations, but comparing GFP that is not side by side is not always easy. Overall, I’m wondering if GFP quantification data should be primary and images backing that up (sometimes in supplemental). Would this help create room in figures to help with legible presentation?

**Our response**: We apologise for the lack of vhl-/- comparison. We have now added a vhl -/- figure close to the double mutants’ in order to improve the comparison with each group (Figure 3E-E’).

Figure 4. Were all images taken then genotyped? Seems that levels of GFP would be difficult to simply sort and verify. These details are not in methods.

**Our response**: Yes, they were. We have initially sorted triples mutants from double mutants and vhl mutants by looking at the GFP intensity. In particular, among all the phenotypic differences observed, the brightness at the level of the tail has been the main indicator of a putative genotype/phenotype correlation. Finally, genotyping post phenotypic analysis allowed to confirm this genotype/phenotype correlation.

Figure 6. MR was shown to not have a role in rapid non-genomic behaviors that required HPA axis signaling in zebrafish (Lee et al, Genes Brain Behavior 2018). This is contrary to Faught & Vijayan manuscript published at the same time and referenced herein.

**Our response**: We acknowledge that and we apologies for the oversight. We have added the suggested reference in the discussion section (page 18, line 7)

The clarity of the paper will depend on clear definitions and use of terms. For example, GC response can be a lot of things, but I think in the discussion model it refers to GR transcriptional regulation. Suppressed HIF/upregulated HIF is generally tied to the HIF-reporter, so again is a transcriptional activity. In the models in Fig 2, there is no “cortisol” step as in the discussion model, so GC response could mean cortisol release there and maybe does? This should be clear and word choice around glucocorticoids (e.g. cortisol) and GR and GR transcriptional activity should be clear.

**Our response**: We apologise for not making this clear, this has been corrected. Moreover, cortisol step has been added to the graph (Figure 2G).

The final model should be worked up a little bit to not only show what you think is happening but perhaps point to supporting data in figures (again figures need better subpanels to make that easier).

**Our response**: We apologise for not making this clear. The final model has been corrected and improved (Figure 2G). Moreover, more subpanels have been added to figures.

The study uses Betamethasone, a glucocorticoid receptor (GR) agonist that has been used clinically for decades and your group recently (2017) showed can activate HIF. Although this GR agonist primarily interacts with GR there is some minimal crosstalk with mineralocorticoid receptor (MR), but this small crosstalk may have biological relevance.

**Our response**: Even if we cannot exclude it, since it well known that BME has insignificant mineralocorticoid activity (Montgomery et al., 1990; Fromage, 2012), we believe that BME is less likely to trigger any MR-related biological activity. Vice versa, as cortisol can bind both to GR and MR, we have speculated that this small GC-Mr dependent crosstalk with HIF signalling may have biological relevance. (page 14, line4-15 and page 18, line 3-16)

**Reviewer #3:**

This manuscript looks at the interaction between glucocorticoids and the HIF pathway. It generates and employs some useful genetic mutants and presents an important addition to the growing field of integration of different stress pathways on physiology. At times it is rather convoluted and complicated and lacks clear logical explanations at parts. If the authors can present the conclusions in a clearer model, the paper would be much improved.

**Our response:** We apologise for not making this clear, this has been corrected. Finally, the final model has been improved (Figure 2G). Moreover, more subpanels have been added to figures to enhance readability.

The authors use the term “attenuated” to describe the partially elevated HIF signaling in the vhl;arnt1 double mutants, which is a little confusing as it is only attenuated with respect to vhl single mutants and not with respect to the WT groundstate. I know what the authors mean, but I suggest they replace this throughout with the term “moderately upregulated” or similar for clarity.

**Our response:** We have amended the manuscript as suggested. Now, the term attenuated has been changed into moderately upregulated throughout the text. Moreover, to improve data readability, the vhl;arnt1 double mutants’ fkbp5 expression has been removed from figure 2A.

For the statement that “In contrast, when the HIF pathway was suppressed (arnt1-/-), BME
was able to further upregulate mainly the expression of fkbp5” the authors need to show the fold upregulation and associated ANOVA Post-test comparing the upregulation of fkbp5, il6st and pck1 in the arnt1 mutants with and without BME treatment as well as with sibs treated with BME.

**Our response:** We apologise for this oversight and we have amended the manuscript as suggested (page 8, line 21-24, fig 2A’ page 36, fig S2B-D page 44)

Figure 2B’ is confusing as it implies mutation of arnt1 inhibits suppression of Hif. This pathway needs to be presented in a more standard manner i.e Hif--| GC-->GR--| pomca.

Similarly the Figure 2C' complicates matters. I appreciate that in the case of increased Hif signalling, a different mechanism is being employed but this seems to contradict Fig2B’. Could the two be combined into one clear model?

**Our response:** We have amended the manuscript as suggested. Figure 2B’ and 2C’ have been combined into one clear model (Figure 2G).

Upregulation of cyp17a2 in arnt1 mutants is claimed, but not decisively shown. The insitu image does not convincingly demonstrate upregulation – A: DMSO sibs vs DMSO mut: stained area quantification and t test stat analysis.

**Our response:** We apologise for this. We have modified the figure 3D-E (now referred as Fig S3A-H’) by adding a magnified picture of the area of interest to clearly show the upregulation of *cyp17a2* in arnt1 mutants (Fig S3C’) and its downregulation in vhl mutants (Fig S3G’), compared to siblings (Fig S3A’-E’). This result has also been confirmed by quantifying the area covered by the WISH staining in the *arnt1* mutants, compared to arnt1 siblings (Fig S3I-I’). Finally, we have moved this figure to supplemental information in order to make room for key novel data.

Figure 3C’ is too small. I cannot see it properly but it appears that arnt1-/-;vhl-/- double mutants have more egln3, and vegfab than the vhl-/- mutants alone. Is that correct on the graph? That seems odd and contrary to my understanding of the hif pathway logic.

**Our response:** No, it is not. The graph shows that arnt1-/-;vhl-/- double mutants have more egln3, and vegfab than the arnt1;vhl wt siblings, as expected (1^st^ blue bar DMSO treated, 2^nd^ blue bar BME treated).

The CRISPANT approach was developed by Burger et al 2016, not Wu et al 2018. The former should be cited rather than the latter.

**Our response:** Apologies for this oversight. Now it has been properly cited.

Given that the authors have previously shown that GR activates the Hif pathway by degrading VHL (Vettori et al 2017), how do they explain the rescue of vhl mutants by GR mutation, which should be hypostatic to vhl.

**Our response:** This is correct, we have to assume another point of interaction between these two pathways in the zebrafish, that is downstream of vhl, we have made this more clear in the discussion

“Consequently, we speculate that in an arnt1 knock-out scenario, pomca downregulation occurs as a consequence of glucocorticoid induced negative feedback loop (aimed to shutdown cortisol biosynthesis). In contrast, as upregulated HIF levels appear to repress pomca expression, we speculate that this occurs in a way that resembles the GR-mediated negative feedback loop.” – I cannot make sense of this statement and needs to be written with much more clarity. The latter sentence invokes the same feedback loop as for arnt1 knockout.

**Our response:** We apologise for not making this clear, this has been corrected. Now we have explained that sentence in a clearer way (page 16, line 17).
