# Supplementary material for: Bidirectional crosstalk between Hypoxia-Inducible Factor and glucocorticoid signalling in zebrafish larvae
Source: PLoS Genet. 2020 May 7;16(5):e1008757. doi: 10.1371/journal.pgen.1008757 (PMC7237044; doi:10.1371/journal.pgen.1008757)
Supplement: Attachment — Submitted filename: Response to reviwers_DMFvE_22-03-2020.docx [file pgen.1008757.s009.docx]

**Comments to the Authors: 22/03/2020
Please note here if the review is uploaded as an attachment.**

**Reviewer #1:**

The authors have addressed most of the issues that I raised and have significantly improved the manuscript. However, I still have a few concerns, which mainly concern the data presented in Figure 2, the description of these results and the part of the Discussion that deals with these results. Most importantly, I think the authors should be more careful with their interpretation of the data, which is at times speculative.

- To my big surprise, the data in Fig.2A’ are different from the results that were originally presented for this experiment. 1) Why were these data substituted? 2) And why were the data from the double mutant removed?

**Our response**: 1) In addressing a number of comments from reviewer #3, we had to redo a few qPCRs and we felt that this was more reliable. 2) The RTqPCR data on moderate upregulated HIF levels’ fkbp5 expression (central graph in figure 2A) has been removed in order to improve the clarity of the manuscript and to allow a more direct and clear comparison between an upregulated and a repressed HIF signalling scenario. The previous manuscript was rich in detail, but we felt that these *-often complex-* details led to confusion and risked losing sight of the main messages. This was something that reviewers brought in the first round of reviews. We believe is easier understand and compare the data when the focus is on extreme scenarios (qPCR, WSH and cortisol quantification).

- The authors use the qPCR data to prove that HIF signaling induces a glucocorticoid-resistant state. However, I do not think these data are entirely convincing, because the glucocorticoid response in the siblings is highly variable, as well as the basal expression levels in the mutant (why for example the fold change upon BME treatment in the (allegedly glucocorticoid resistant) vhl mutant is higher than in wild type). The authors should at least be more careful with statements about the glucocorticoid resistant state since it is solely based on these data.

**Our response:** We acknowledge your point. We believe this is because BME, being a synthetic GR agonist, acts downstream of the HPI axis and directly binds to GR. Since GR activity is strictly tied to Fkbp5 expression, as part of an intracellular ultra-short negative feedback loop, we quantified its expression both in the *vhl* and the *arnt1* mutant line. Interestingly, in DMSO treated *vhl* mutants, we have observed a strong downregulation not only of *pomca*, but also of *fkbp5*, coupled to decreased cortisol levels. For these reasons, we have speculated that in this scenario, BME administration can overcome the HIF-mediated inhibition of the stress response (*pomca* expression), by directly acting on GR and can mildly stimulate GC-GR response (i.e upregulated *fkbp5*), compared to DMSO treated vhl mutants (**P=0,0022). However, the upregulated BME treated *vhl* mutants’ *fkbp5* levels (fold change= 2,1; P=0.891) are not significantly higher than the DMSO treated wildtypes ones.

- In Fig.2G, the arrow from low pomca to high cortisol in the arnt1 mutant is still difficult to explain, although the increased GC/GR response could play a role, as the authors suggest. They should be a bit more careful here as well, and at least make clear that the combination high cortisol/low pomca is very rare and difficult to explain (and that this combination may change over the course of development).

**Our response:** We acknowledge your point and we have amended the manuscript as suggested (page 34, line 37-40)

- The altered response to BME should be incorporated in the top panel of Fig.2G, by drawing a direct arrow from HIF to GC/GR response (with BME next to that arrow). Now it looks like the increased response to BME is a result of HIF acting through pomca and cortisol.

**Our response:** We have changed the text to make our statements more careful. We changed “representative model” to “a speculative model”. We have added a statement that acknowledges there may be more direct effect of HIF on GC/GR in the figure legend. We preferred this to adding another arrow as the presence of too many arrows makes the figure difficult to understand. For this reason, we strongly prefer not to add BME in the upper panel of the 2G as this would be confusing since the side panel (DMSO) indicates there is no BME added. (page 34, line 27)

Minor points:

- Page 6, line 18: Hif-1β is not a nuclear receptor.

**Our response**: We apologise for this oversight, this has been corrected (Page 6, line 16)

- Page 7, line 25: please mention that the levels are still higher than the wild type, and preferably also why these levels are not shown in Fig.1K (I assume because they are undetectable?).

**Our response**: We apologise for not making this clear. We have amended the manuscript as suggested. Yes, the wildtypes *phd3:eGFP* levels are not shown because they are undetectable. (Page 7, line 27-29)

- In the figures, sometime controls are labeled ‘sibs’ and sometimes ‘wt’. If there is no particular reason for this difference, please make it consistent.

**Our response**: We apologise for this oversight; this has been corrected.

**Reviewer #2:**

The revised manuscript by March et al has been vastly improved by the responses of the authors to the reviewers. There remain some minor typos.

Example from abstract "...both the glucocorticoid receptor (Gr) responsiveness and the endogenous cortisol..." could be "...both glucocorticoid receptor (Gr) responsiveness and endogenous cortisol...".

Also, in reference to Gr and Mr the authors state "Together they act as a transcription factor, which can function in either a genomic or in non-genomic way". This is confusing because in their role as a transcription factor that is by definition a genomic response by changing transcription. However, both particularly GR, are likely involved in other cell signaling that does not involve it's conventional role as a transcription factor and this is referred to as non-genomic.

Overall, this paper which by its very nature can be complicated is vastly improved. Thank you for your efforts.

**Our response:** We accept all these points and have addressed them accordingly. We agree that the naming is poorly chosen. We would only stress the point that in the GR/MR field this is a well-established way of describing these activities (Stahn and Buttgereit, 2008; Mitre-Aguilar, Cabrera-Quintero and Zentella-Dehesa, 2015; Facchinello *et al.*, 2017; Laranjinha *et al.*, 2018; Panettieri *et al.*, 2019; Strehl *et al.*, 2019). For this reason, we believe that introducing yet a further designation for these activities would be also confusing. Therefore, we hope that is fine with you if we stick with them. (page 3, line 1)
